# Cognitive profiles in older males and females

**DOI:** 10.1038/s41598-021-84134-8

**Published:** 2021-03-22

**Authors:** C. Jockwitz, L. Wiersch, J. Stumme, S. Caspers

**Affiliations:** 1 Institute of Neuroscience and Medicine (INM-1), Research Centre Jülich, Jülich, Germany; 2 Institute for Anatomy I, Medical Faculty & University Hospital Düsseldorf, Heinrich Heine University Düsseldorf, Düsseldorf, Germany; 3 Institute of Neuroscience and Medicine (INM-7), Research Centre Jülich, Jülich, Germany

**Keywords:** Human behaviour, Cognitive ageing, Ageing

## Abstract

Males and females are subject to differences in cognitive processing strategies, i.e. the way males and females solve cognitive tasks. So far primarily reported for younger adults, this seems to be especially important in older adults, who also show sex differences in cognitive impairments. Therefore, the aim of the current study was to examine the older adult population with respect to cognitive profiles derived from a large variety of cognitive functions. Using an exploratory component analysis with consecutive confirmatory factor analysis in a sample of 676 older adults, neuropsychological performance data in a variety of cognitive domains was decomposed into cognitive components. A general cognitive profile based on the whole group fits unequally well on the two sexes. Importantly, cognitive profiles based on either males or females differ in terms of their composition of cognitive components, i.e. three components in males versus four components in females, with a generally better model fit in females. Thus, related to the established differences in processing styles between males and females the current study found a rather decomposed (or local) cognitive profile in females while males seem to show a holistic (or global) cognitive profile, with more interrelations between different cognitive functions.

## Introduction

There has been a longstanding debate about whether males and females differ in terms of cognitive abilities. Males are often supposed to outperform females in visual spatial tasks, while females outperform males in terms of verbal and episodic memory tasks^1–6^. While these sex stereotypes are well accepted in our society^7^, there is a non-negligible amount of studies showing exactly the opposite, namely that men and women do not differ in most of the cognitive tasks, also referred to as the “Gender Similarity Hypothesis”^8,9^. That is, cognitive performance differences on average show an effect size of d = 0.22 (range: 0.05–0.57) which is interpreted as rather small differences. Using a meta-synthesis approach, Zell et al.^10^, however, concluded that sex differences in terms of psychological and cognitive variables is rather small but stable across ages, generations and cultures.

Besides investigating sex differences in absolute cognitive performance outcome measures (i.e. females remember more words from a word list as compared to males), recent studies rather focussed on sex differences in cognitive processing styles, i.e. the way males and females solve a given cognitive task^11,12^. For example, in spatial navigation tests, females were found to use local landmarks to find a specific route, while males rather construct cognitive maps of the environment^11,13,14^. Interestingly, when males and females are instructed to actively choose a landmark-based style, females outperform males in this task^13^. Similarly, in a verbal fluency task, Weiss et al.^15^ as well as Lanting et al.^16^ showed that the males’ processing strategy is typically characterized by a systematic and extensive scan of the word space of a given category before moving to the next one (e.g. listing jobs, males would first list all jobs within a hospital, then within an office etc.). In contrast, females switch more often between different categories. Changing the instructions, i.e. inducing more switches between categories, led to superior performance of females^12^. Thus, based on previous research investigating specific cognitive tasks, it has been established that males and females use different cognitive processing strategies: Males seem to use a rather holistic processing style with a focus on global aspects of the task (i.e. having in mind the whole map of a city when performing a spatial navigation task). Females instead use a decomposed processing style with a focus on more local aspects of the task (i.e. remember more details of a given word list). Similar sex differences in terms of a global versus local focus have been found for other tasks such as mental rotation tasks^17^, number-comparison-task^18^ and Navon paradigms^19^.

Although sex-related differences in cognitive processing styles do not necessarily result in differences in performance in everyday life, i.e. males and females perform equally good in an everyday multitasking paradigm^20^, they give rise to the question of whether males and females do not only differ in single cognitive abilities. Rather, the two sexes might generally differ in the overall composition of their cognitive abilities. So far, studies mostly focus on cognitive profiles that are predefined based on specific cognitive theories or derived from data-driven approaches (e.g. principal component analyses)^21–25^. For example, single cognitive abilities are often categorized into cognitive domains, such as attention, memory and executive functions, based on correlations between performance in the tasks administered^21^. Performance within the cognitive domains, then, together represent cognitive profiles. Typically, such approaches are based on an entire group including both, males and females. However, whether these cognitive components and profiles reflect the cognitive architecture equally well across the two sexes remains unclear. The relation between performance in distinct cognitive tasks might be differentially related to each other in males and females and therefore might form different sex-specific cognitive profiles.

Particularly interesting in this aspect is the older adult population, since sex differences in cognitive performance were found to persist until late adulthood and might even lead to differences in cognitive impairments during older age and disease^2,22,23^. So far, the majority of studies investigate cognitive performance during aging while correcting for sex differences. Averaged over the two sexes, cognitive performance decline is well established during the aging process^24–27^ with a significant decline starting in the mid 50’s^24^, especially in the domains of executive functions, working memory and episodic memory. However, previous studies not only showed that sex-differences in cognitive performance persist until late adulthood^2,22,23^, they also reported unbalanced prevalence in neurodegenerative diseases that are accompanied by different cognitive impairments, i.e. males rather suffer from MCI and Parkinson’s disease, while females are more often affected by Alzheimer’s disease^28,29^. Potentially, different interrelations between cognitive functions might explain parts of these different age-related trajectories and therefore depict a promising research topic. To examine this, the current study took advantage of a large older adult population of males and females between 55 and 85 years from the 1000BRAINS cohort, matched for age and education, and examined the sex-specificity of cognitive profiles based on a large variety of neuropsychological functions. Using a data-driven approach, neuropsychological test performance was first decomposed into cognitive components. Afterwards the different component solutions were statistically compared between the two sexes. Based on the sex-specific strategies found when investigating specific cognitive tasks (i.e. global versus local processing strategies), we would expect these differences to be also reflected in sex-specific cognitive profiles.

## Methods

### Subjects

Subjects included in the current study were drawn from 1000BRAINS^31^, a population-based epidemiological cohort study, recruited from the Heinz-Nixdorf recall study that has been conducted in the Ruhr area in Germany^32^. Along the line of being population-based, exclusion from the study was based on eligibility for MR measurements for scientific purposes. From the initial cohort of 1314 subjects, 968 subjects being 55 years and older were selected to assess the older adult population. 20 subjects had to be excluded due to missing variables of interest for the current study (DemTect^33^: n = 18; or information on education: n = 2). Furthermore, subjects missing more than three values of the neuropsychological assessment (n = 2; for all other subjects missing values (ranging from 0 to 2.1% depending on the test) were replaced by the median of the respective age- (< 60; 60–64; 65–69; 70–74; 75–79; < 79) and sex-group. Subjects representing outliers (n = 83; outliermax > mean + 3*SD; outliermin < mean − 3*SD) in at least one of the cognitive variables were removed from the dataset. To establish similar demographic conditions in the two sex groups, propensity score matching (method = "nearest", caliper = 0.25; implemented in R: matchit, version 3.0.3) was used to match males and females for age and education (measured by ISCED^30^) which resulted in a final sample size of 676 subjects between 55 and 87 years of age: 338 males with a mean age of 66.9 years (SD = 6.7) and a mean ISCED score of 6.3 (SD = 1.74) and 338 females with a mean age of 66 years (SD = 6.5) and a mean ISCED score of 6.1 (SD = 1.86). All participants gave written informed consent before participating in 1000BRAINS. All experiments were performed in accordance with relevant named guidelines and regulations. The study protocol was approved by the local Ethics Committee of the University of Essen.

### Neuropsychological assessment

All subjects underwent intensive neuropsychological testing during their participation in 1000BRAINS^31^. In total, 16 different cognitive functions, namely selective attention, processing speed, problem solving, concept shifting, susceptibility to interference, figural fluency, phonematic and semantic verbal fluency, vocabulary, verbal episodic memory, figural memory, visual-, visual-spatial- and verbal short-term/working memory were assessed. For cognitive functions and tests used, as well as raw mean scores for males and females, see Table 1.

**Table 1:** Descriptives of neuropsychological variables including cognitive functions, tasks used, mean and standard deviation (SD) and Min; Max values, T value of group comparison with corresponding *p* value and effect size measured with Cohen′s d.

Cognitive Function	Test description	Females: mean ± SD (Min; Max)	Males: mean ± SD (Min; Max)	T-value	*p-* value	Cohen's d
Age		65.99 ± 6.5 (55.2;85.4)	66.87 ± 6.65 (55.1;85.4)	− 1.735	0.083	0.132
DemTect	*DemTect*^33^*: Global cognitive score*	15.55 ± 2.22 (8;18)	14.17 ± 2.36 (8;18)	**7.860**	**0.000**	− **0.587**
ISCED97	*International Classification*^30^*: Education classification*	6.1 ± 1.86 (3;10)	6.29 ± 1.74 (3;10)	− 1.370	0.171	0.109
Problem solving	*Leistungsprüfungssystem 50* + *(Subtest 3)*^34^*: Number of correctly identified non-matching figures among geometrical figures*	20.39 ± 4.71 (8;35)	20.82 ± 5.13 (8;34)	− 1.132	0.258	0.084
Visual STM	*Block-Tapping-Test*^35^*: Number of correctly repeated blocks, forwards*	6.32 ± 1.76 (2;10)	6.57 ± 1.65 (2;10)	− 1.937	0.053	0.154
Visual WM	*Block-Tapping-Test*^35^*: Number of correctly repeated blocks, backwards*	4.69 ± 1.65 (1;10)	5.04 ± 1.7 (0;10)	− **2.738**	**0.006**	**0.208**
VisualSpatial STM	*Visual pattern (Jülich version; similar to* ^36^*): Number of memorized patterns presented in a grid of black and white squares*	7.32 ± 1.7 (4;12)	8.06 ± 1.68 (4;12)	− **5.711**	**0.000**	**0.443**
Verbal STM	*Zahlennachsprechen (from Nürnberger Alters-Inventar*^37^*): Number of correctly repeated digits, forwards*	7.63 ± 1.84 (4;13)	7.66 ± 2.02 (4;13)	− 0.179	0.858	0.013
Verbal WM	*Zahlennachsprechen (from Nürnberger Alters-Inventar*^37^*): Number of correctly repeated digits, backwards*	6.79 ± 1.65 (2;12)	6.87 ± 1.77 (2;12)	− 0.653	0.514	0.049
Figural memory	*Benton-Test*^38^*: Number of errors during free recall of 20 remembered figures*	− 16.33 ± 7.57 (− 40; − 2)	− 16.17 ± 7.56 (− 36; − 1)	− 0.275	0.784	− 0.021
Selective attention	*Alters-Konzentrations-Test*^39^*: Time(s) to recognize target figures among distractors*	− 33.54 ± 8.74 (− 64.78; − 17)	− 33.66 ± 8.38 (− 65.87; − 18.22)	0.183	0.855	0.014
Interference	*Farb-Wort-Interferenztest (Jülich version; similar to: Bäumler*^40^*; Stroop*^41^*): Time(s) to name ink color of words with color meaning* *but printed in a different color (subtracted by the time(s) to read color words)*	− 39.63 ± 16.64 (− 110.6; − 9.47)	− 43.36 ± 17.58 (− 109.97; − 3.66)	**2.833**	**0.005**	**0.212**
Figural fluency	*Fünf-Punkte-Test (Jülich version; similar to: Regard *et al*.*^42^*): Number of unique drawn patterns by connecting five points in 3 min*	26.15 ± 6.89 (4;44)	26.38 ± 7.22 (11;49)	− 0.425	0.671	0.032
Episodic memory	*Verbaler Gedächtnistest*^43^*: Number of free recalled words in five trials from a list containing 15 words*	45.76 ± 10.05 (2;66)	38.61 ± 10.01 (6;65)	**9.262**	**0.000**	− **0.714**
Phonematic fluency	*Regensburger Wortflüssigkeitstest*^44^*: Number of produced words beginning with the letter “B”*	19.32 ± 6.04 (4;37)	17.49 ± 5.93 (5;37)	**3.992**	**0.000**	− **0.310**
Semantic fluency	*Regensburger Wortflüssigkeitstest*^44^*: Number of produced words belonging to the category “jobs”*	24.47 ± 6.19 (11;44)	23.39 ± 6.76 (6;43)	**2.153**	**0.032**	− **0.159**
Processing speed	*Trail Making Test (taken from CERAD-Plus*^45^*): Time(sec.) to connect randomly arranged digits in ascending order*	− 38.62 ± 11.71 (− 79.41; − 16.06)	− 40.22 ± 13 (− 84.18; − 16.13)	1.677	0.094	0.123
Concept shifting	*Trail Making Test (taken from CERAD-Plus*^45^*): Time(sec.) to alternately connect letters and numbers in ascending order (TMT B), then calculating:* *TMT B-TMT A*	− 48.71 ± 28.33 (− 183.44; − 1.78)	− 54.28 ± 32.46 (− 166.6;0.67)	**2.375**	**0.018**	**0.171**
Vocabulary	*Wortschatztest*^46^*: Number of correctly identified real words among five pseudo-words*	30.96 ± 4.34 (16;40)	30.8 ± 4.17 (16;40)	0.493	0.622	− 0.039

Values written in bold indicate significant differences between groups (*p* < .05). STM = short-term memory, WM = working memory.

### Statistical analyses

First, sex differences in cognitive performance were examined for the different cognitive functions assessed in 16 different neuropsychological tests using Independent Sample T-Tests. Effect sizes were calculated using Cohen’s d. Afterwards, we calculated z-scores for each variable followed by Pearson correlations between all neuropsychological variables included in the current analysis for the whole group, as well as for males and females separately.

**Figure 1:**
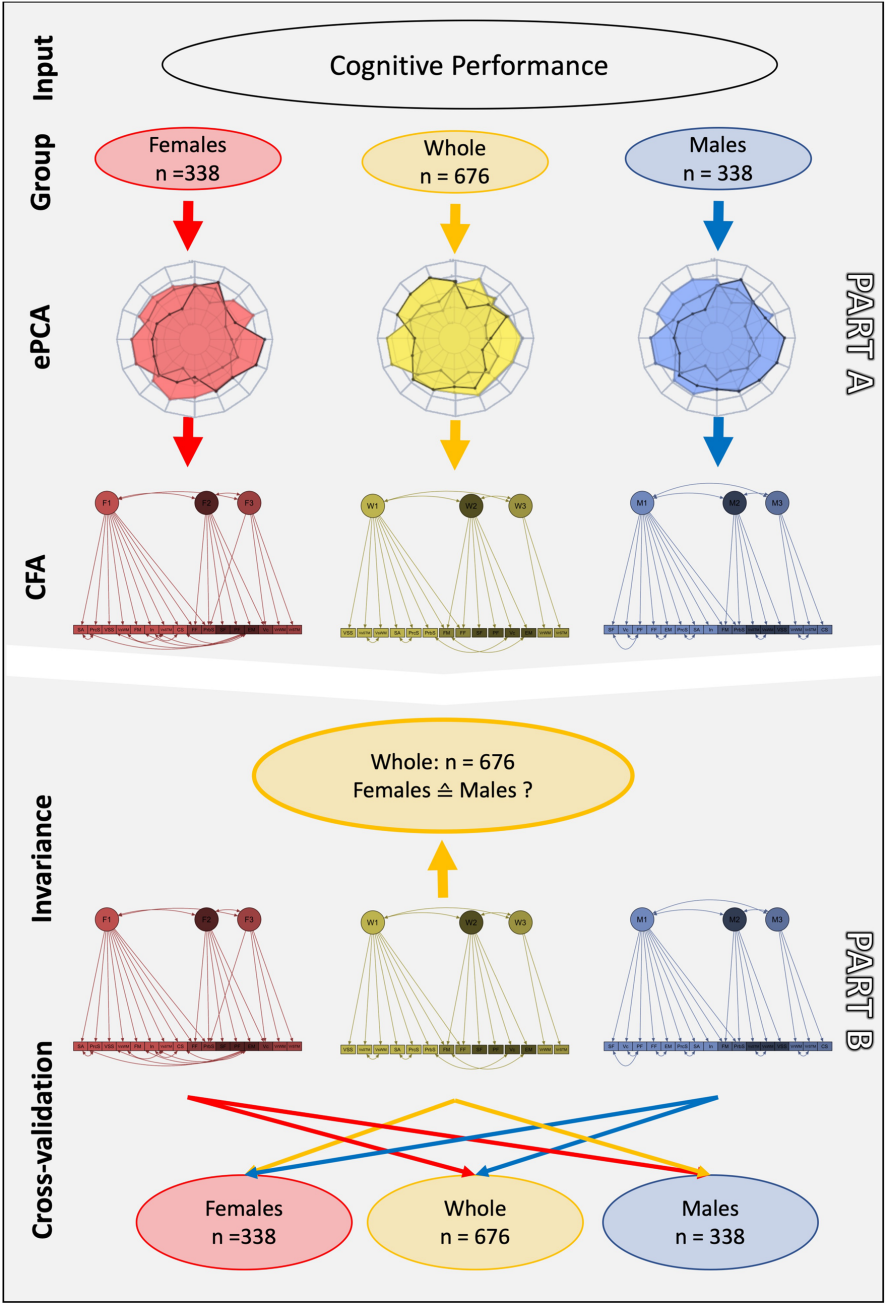
Flowchart presenting the study design.

The major research question in this study concerned whether males and females would show different cognitive profiles, i.e. different compositions of cognitive components. To investigate this, we divided our analyses in two parts (for an overview of analyses, see Fig. 1, part A and part B). In the first part (part A), we extracted cognitive components for both, the whole group (n = 676) including males and females, as it is commonly done in research investigating cognitive performance (e.g. see^46–51^, as well as for males (n = 338) and females (n = 338) separately to identify commonalities as well as differences in cognitive profiles between the two sexes. For all the groups (whole, males and females) a two-step approach was applied:

1. *Data reduction* We reduced the cognitive performance data into independent cognitive components by using exploratory principal component analysis (ePCA) with Varimax rotation (implemented in the “psych” package, R Studio), as one of the most commonly used technique for data reduction^52^. The number of extracted components was based on the eigenvalue criterion (eigenvalue > 1). This resulted in three independent data-driven component solutions: whole ePCA based on the whole group, male ePCA within males only, females ePCA within females.
2. *Component solution validation within respective groups* To validate the obtained component solutions in their respective group (whole ePCA, male ePCA, females ePCA), a confirmatory factor analysis (CFA, implemented in the “lavaan” package, R Studio) was set up with Maximum Likelihood estimator with robust standard errors and a Satorra-Bentler scaled test statistic. In detail, each component solution represents a measurement model that is composed of a specific number of cognitive components, with each including a specific number of cognitive performance tests. In the current study, we based the measurement models on the component solutions obtained by ePCA and included all cognitive performance tests with a component loading of at least 0.4^53^.

To examine model fit of the respective ePCA’s, we used comparative fit index (CFI), tucker lewis index (TLI), root mean square error of approximation (RMSEA) and standardized root mean square residual (SRMR). Quality of model fit was assessed based on frequently reported fit indices indicating excellent model fit at CFI > 0.95, TLI > 0.95, RMSEA =< 0.06, SRMR < 0.09^54,55^. All initial models were subsequently refined to increase model fit: From the initial model, we first modelled residual covariances (included when residual covariances were > 0.1) between variables and components, and afterwards, removed non-significant variables from the model, if present.

After this measurement model configuration, we attempted to validate the established models across the two sexes (Fig. 1, part B). To do so, we first examined measurement invariance for all three models (whole CFA, male CFA, female CFA). Measurement invariance addresses the question whether a scale measures the same attribute in different groups of subjects. Hence, in the current study, measurement invariance would test whether the different cognitive component solutions, i.e. cognitive profiles would be the same across males and females. Measurement invariance was tested with the following aspects: (1) configural invariance: the measurement models derived from the CFA would fit equally well in males and females (same data structure across variables); (2) loading invariance: loadings of variables onto a cognitive component would be the same for males and females (groups have the same factor loadings); (3) intercept invariance: males and females would show the same intercept on the measured variables (groups have same intercepts of the observed variables); (4) mean invariance: males and females would show the same means on the measured variables (groups have the same means across the observed variables). In a second step, we applied a strict cross-validation by applying the sex-specific models to the other sex group only to test whether the male component solution would also obtain a good fit in females and vice versa. Model fit changes across the models were considered as significant with a change in CFI > 0.01^56^ and a significant likelihood chi square difference test (*p* < 0.05).

## Results

The current study assessed sex differences in cognitive profiles between older males and females based on a large battery of cognitive tests assessing attention, memory, executive and language functions. Differences in performance between males and females were already observed at single test level in several of the 16 neuropsychological tests used in the current study. For example, males performed significantly better in tasks requiring visual and visual-spatial abilities, e.g. visual-spatial memory, whereas females performed better in tasks requiring verbal abilities, such as episodic learning, phonematic and semantic fluency (see Table 1).

**Figure 2:**
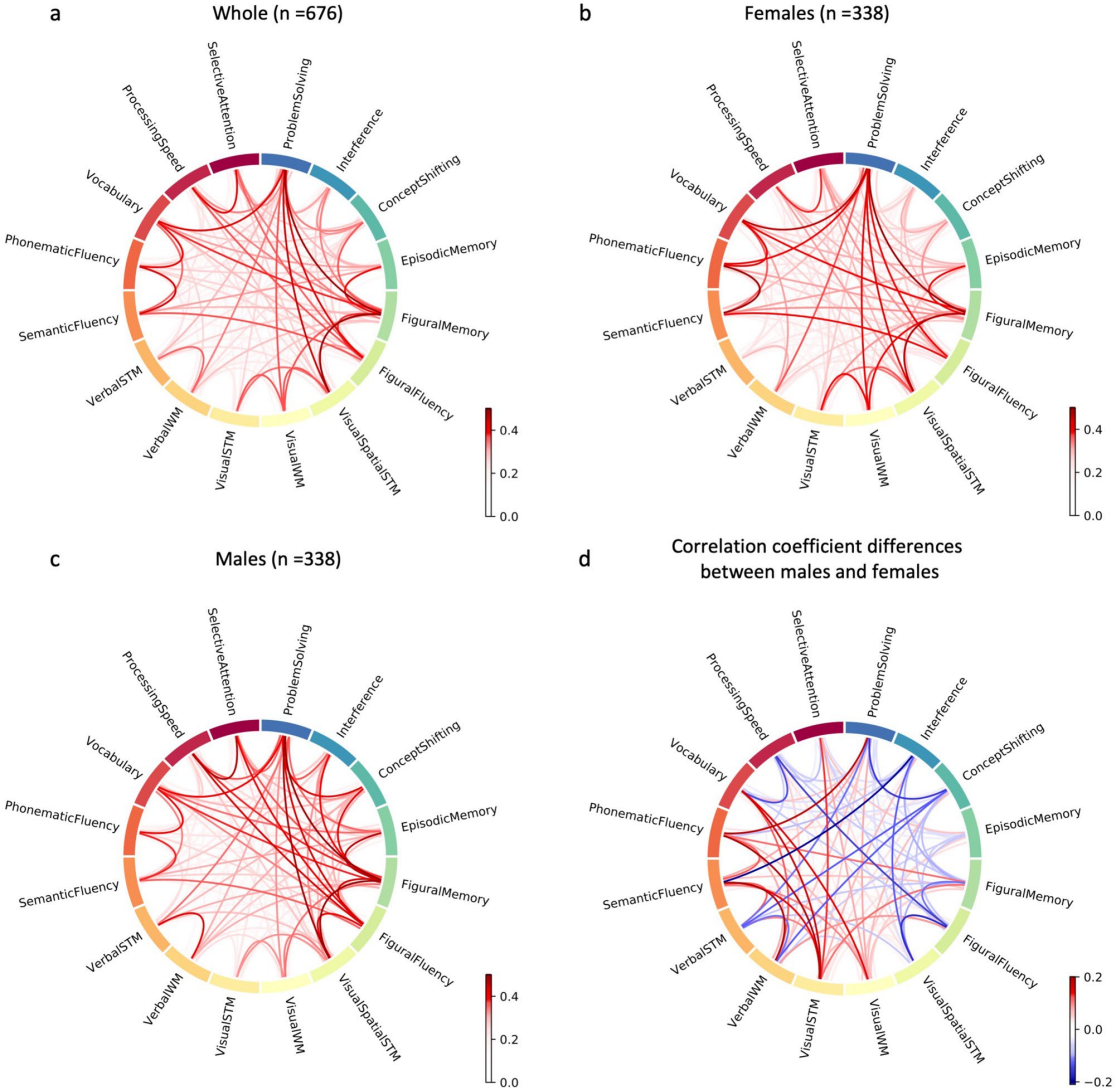
Chord diagrams of correlations between cognitive performance tests: (**a**) whole group, (**b**) females and (**c**) males, (**d**) differences in correlation coefficients between males and females: blue = males > females, red = females > males.

Investigating intercorrelations between cognitive performance scores of the different cognitive functions revealed a second interesting and important observation: While we overall found high intercorrelations between the assessed cognitive performances, these intercorrelations do not seem to be identical in males and females, already hinting at differences in cognitive profiles for the two sexes (for chord diagrams for the whole group, males and females separately as well differences between males and females, see Fig. 2, for Pearson correlation values between cognitive task, see Supplement, Tables S1–S3). Sex differences in cognitive performance correlations are shown in Fig. 2d. Noticeably, females show higher correlations between verbal and non-verbal test performance while males show higher correlations between verbal, non-verbal and executive functions (e.g. interference, concept shifting and problem solving).

### Principal component analyses and confirmatory factor analyses for the whole group and males and females separately

Based on the correlations between cognitive performance tests, ePCA was applied to individual cognitive performance measurements of the whole group as well as males and females separately. Extraction of components was based on the eigenvalue criterion (eigenvalue > 1, see Supplement, Table S4). Three components were extracted for the whole group (eigenvalues: 4.94, 1.48, 1.19) as well as when assessing males only (eigenvalues: 5.08, 1.58, 1.25). Regarding females only, the optimal component solution consisted of four cognitive components (eigenvalues: 4.97, 1.36, 1.09, 1.02). For all eigenvalues, see Supplement, Table S4.

**Figure 3:**
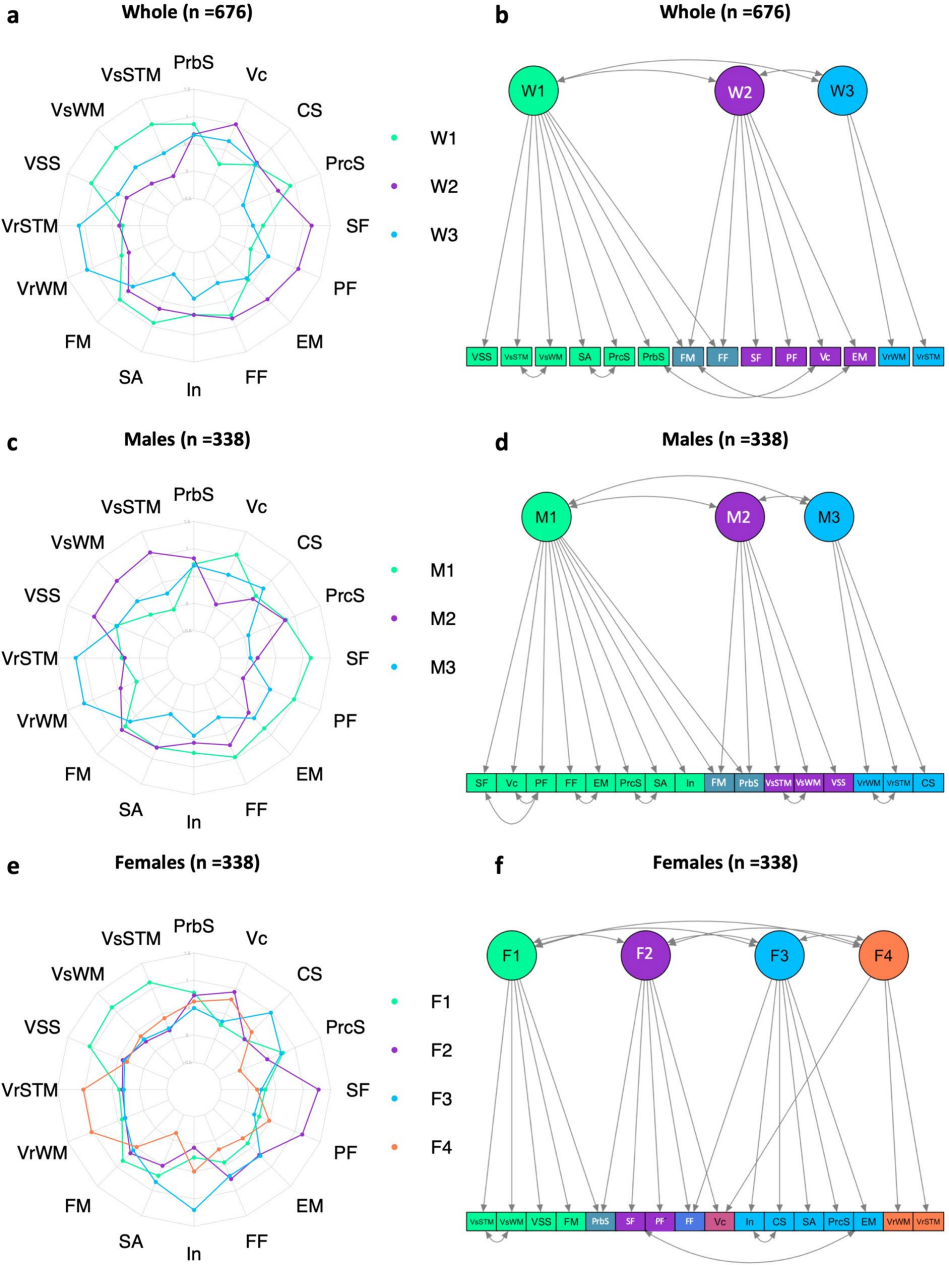
Exploratory Principal Component Analysis (ePCA) and Confirmatory Factor Analysis (CFA): (**a**,**c**,**e**): ePCA for the whole group, males and females. (**b**,**d**,**f**): CFA for the whole group, males and females.* PrbS* problem solving, *VsSTM* visual spatial short-term memory, *VsWM* visual spatial working memory, *VSS* visual working memory, *VrSTM* verbal short-term memory, *VrWM* verbal working memory, *FM* figural memory, *SA* selective attention, *In* interference, *FF* figural fluency, *EM* episodic memory, *PF* phonemic fluency, *ST* semantic fluency, *PrcS* processing speed, *CS* concept shifting, *Vc* vocabulary.

For the whole group, the extracted components were dominated by the following functions: The first component covered a variety of non-verbal cognitive functions such as visual working memory, attention, executive functions and memory. The second component included fluency as well as memory. The third component was dominated by verbal functions, such as verbal working memory and vocabulary (Fig. 3a, for component loadings of all groups, see Supplement, Table S5). Afterwards, we extracted fit values for the PCA-derived three-component model using CFA. All variables were found to significantly contribute to the components (> 0.4), However, fit values of the initial model were not to be considered as of sufficient quality (CFI = 0.894; TLI = 0.866; RMSEA = 0.07, SRMR = 0.053). After model refinement via inclusion of residual covariances and exclusion of non-significant variables, the model improved significantly, but did not reach the threshold for being an excellent model in all fit indices (CFI = 0.941; TLI = 0.921; RMSEA = 0.054, SRMR = 0.045). The resulting model is shown in Fig. 3b (for results of the CFA for all groups, see Supplement, Table S6 and S7).

The male model (Fig. 3c,d), also a three-component model, consisted of a first component that included fluency, memory, attention and executive function, a second component that was dominated by visual working memory and executive functions and a third component including verbal working memory and executive functions. The initial male model revealed fit values not to be considered as of sufficient quality (CFI = 0.883, TLI = 0.854, RMSEA = 0.071, SRMR = 0.06). After additional refinement, the male model fitted on males revealed fit indices of: CFI = 0.94, TLI = 0.923, RMSEA = 0.051, SRMR = 0.049. This is a significant increase in model fit although it still does not reach the threshold for being an excellent model.

The female component solution (Fig. 3e,f) revealed one component dominated by visual memory and working memory, a second component including fluency and vocabulary and executive functions, a third component that consisted of executive functions and memory and a fourth component including verbal working memory and vocabulary (for variable loading on the different components, see Supplement, Table S5). The initial female model fitted on females revealed fit indices of: CFI = 0.964, TLI = 0.953, RMSEA = 0.037, SRMR = 0.039. Although this model fulfilled the requirements for being an excellent model, we additionally refined the model by the same conditions we did before. This resulted in an additional significant increase in model fit (CFI = 0.984, TLI = 0.979, RMSEA = 0.025, SRMR = 0.034).

Taken together, the investigation of data-driven cognitive components in the three groups (whole group, males and females) hint at different compositions of cognitive components in older males and females (i.e. three versus four components, for an additional overview of three versus four component solutions for the whole group, males and females, see Supplement, Figure S1). Comparing these to the again slightly different component solution derived from the whole group (including both males and females) raises the question of whether these so far descriptively compared differences would be statistically meaningful, which was tested afterwards.

### Measurement invariance and cross-validation

In the second part of the study (Fig. 1, part B), we addressed the distinctiveness of cognitive components between males and females by using measurement invariance and cross-validation. In detail, we started with the component solution that was derived from the whole group (including males and females) and tested whether this whole group cognitive component solution would be statistically the same across males and females, i.e. invariant (for CFA model estimates, see Supplement, Table S8). Model fit indices did not reach the threshold for measurement invariance in terms of configural model (i.e. same data structure: CFI = 0.939, RMSEA = 0.055) and loading invariance (i.e. same factor loadings: CFI = 0.938, RMSEA = 0.053), it did even less so in the intercept (i.e. same intercept: CFI = 0.893, RSMEA = 0.067) and means invariance (i.e. same means: CFI = 0.871, RSMEA = 0.073). Thus, the cognitive component solution derived from the whole group, as it is often done in research investigating cognitive performance, does not seem to be completely generalizable over males and females. This, in turn, leads to the question which group (males or females) would fit better to the whole group component solution. While the model fit increased when the whole group model was applied to females only (whole group: CFI = 0.941; TLI = 0.921; RMSEA = 0.054, SRMR = 0.045; females: CFI = 0.962, TLI = 0.949, RSMEA = 0.043, SRMR = 0.043), it significantly decreased when investigating males only (CFI = 0.919, TLI = 0.892, RSMEA = 0.065, SRMR = 0.058). Thus, the current results indicate that the overall composition of cognitive components derived from the whole group is better suited for the female group as compared to the male group.

In a final cross validation, we applied the different cognitive component models obtained by either the whole group, males or females to the other groups, e.g. male component solution fitted onto the female group and vice versa (for fit indices, see Table 2). Applying the whole group model to males and females separately revealed an excellent model fit for the female group and a worse model fit for the male group. Applying the female cognitive component model to the male group reveals an overall insufficient model fit, which underpins the results obtained by the examination of measurement invariance. In turn, applying the male component model to the female group revealed a reasonable fit, with excellent fit indices. Thus, while males’ cognitive performance does not seem to be sufficiently explained by the female model, female’s cognitive performance can be sufficiently composed into both, male and female component solutions, with a slightly better fit of the female cognitive component model. Nevertheless, applying the male component solution to the female group revealed high covariances between the components (> 1), which indicates collinearity between the components. Thus, the validation of the component solutions indicate that separate cognitive component solutions might better describe a cognitive profile as compared to a common component solution.

**Table 2:** Model fit indices for male and female refined models applied to the different groups.

Model	Group	X2	CFI	TLI	RMSEA	SRMR
WHOLE	WHOLE	**201.647**	0.941	0.921	**0.054**	**0.045**
WHOLE	MALES	**165.421**	0.919	0.892	0.065	**0.058**
WHOLE	FEMALES	**109.756**	**0.962**	0.949	**0.043**	**0.043**
MALE	WHOLE	**221.151**	**0.95**	0.935	**0.045**	**0.04**
MALE	MALES	**175.919**	0.94	0.923	**0.051**	**0.049**
MALE	FEMALES	**142.817**	**0.959**	0.947	**0.04**	**0.043**
FEMALE	WHOLE	**247.081**	0.939	0.921	**0.05**	**0.04**
FEMALE	MALES	**207.401**	0.917	0.891	0.061	**0.051**
FEMALE	FEMALES	**117.601**	**0.979**	**0.972**	**0.029**	**0.036**

Values in bold reach the threshold for being an excellent model.

## Discussion

Using a data-driven approach, the current study examined sex-specific cognitive profiles based on a large variety of cognitive functions in older males and females. Our results show that a general model consisting of cognitive components that combine numerous cognitive tasks calculated based on the whole group (including both, males and females) fit unequally well on the two sexes. Males and females differ in terms of their composition of cognitive components, i.e. three components in males versus four components in females, with a generally better model fit in females. Thus, the current study found a rather decomposed (or local) cognitive profile in females while males seem to show a holistic (or global) cognitive profile, with more interrelations between different cognitive functions.

In a first step, we systematically examined sex differences in 16 different cognitive functions, namely selective attention, processing speed, problem solving, concept shifting, susceptibility to interference, figural fluency, phonematic and semantic verbal fluency, vocabulary, verbal episodic memory, figural memory, visual-, visual-spatial- and verbal short-term/working memory. We showed that older women perform better in verbal fluency, verbal episodic memory, processing speed and interference while older men significantly performed better on visual and visual-spatial working memory tasks. Importantly, these differences were rather small with only visual short-term memory and episodic memory showing medium effect sizes. Hence, the results are in line with a large amount of previous studies showing that males and females differ in some but not all cognitive functions and that these differences tend to be small^5,9,10^. Thus, in normal older adults, we were able to show that those tasks requiring high verbal versus visuospatial processing show the largest sex differences.

Further, de Frias et al.^2^ presented long-term sex differences in cognitive performance in a sample of adults with an age range from 35 to 80 years (at baseline). Over a period of ten years, women remained better in tasks assessing verbal episodic memory and verbal fluency, while men outperformed women in tasks assessing visuospatial functions. Additionally, and in line with Maitland et al.^57^ and Pauls et al.^58^ we showed that sex differences, especially in the verbal versus spatial domains remain stable even in older ages. Thus, the current study adds to the notion that, even in later decades of life, sex differences in verbal versus visuospatial cognitive functions persist.

The observed sex differences in cognitive performance might be due to different processing styles to solve cognitive problems. Men usually inspect new scenes in a more ‘global’ way (e.g. having in mind the whole map in a spatial navigation task), while women usually prefer to inspect tasks more locally (i.e. remember more details of a given word list)^18,19,59^. This might explain why men outperform women with respect to visual-spatial tasks and why women perform better in verbal episodic memory. Based on these task specific differences between the two sexes, the main goal was to investigate whether we could extend this global versus local phenomenon, to cognitive profiles in males and females, i.e. the relations between cognitive abilities. Using a data-driven ePCA we revealed a three-component solution for the whole including: (1) a non-verbal component composed of tasks including attention, executive functions and (working-) memory, (2) a mixed component including verbal and non-verbal fluency and memory functions and (3) a verbal short-term/working memory. This data-driven cognitive component solution shows the high complexity between cognitive functions, i.e. verbal fluency tasks require a large memory span and vice versa, an observation that has been found to be impaired in amnestic mild cognitive performance^60^. It furthermore shows that cognitive components do not necessarily comply with the classical theory-driven cognitive domains of attention, executive functions, working and episodic memory and language functions, an observation that has already been described by Harvey^21^.

Noticeably, CFA was used to examine the model fit indices of this component analysis and whether this component solution fit equally well to males and females. The overall model shows an acceptable, although not excellent fit (CFI > 0.95)^54,55^ for the whole group (even after refinement of the model by including residual covariances between cognitive variables and exclusion of non-significant variables). When examining measurement invariance between the two sexes, thus whether a cognitive profile would fit equally well to males and females, we again found an acceptable but not excellent model fit already in the configural model (composition of the components), with further significant decreases when it comes to mean and intercept invariances. While some fit values do not differ from previous results obtained by Siedlecki et al.^22^, who interpreted a CFI value of 0.941 as being acceptable, they are low as compared to other studies investigating measurement invariance in cognitive or psychological profiles between, e.g. healthy adults and Alzheimer patients or using longitudinal models of sex differences over the whole adulthood^61,62^. These differences in model fit to the aforementioned studies might be due to differences in neuropsychological tests used or differences in group characteristics. In the current sample, normal older adults were examined that were matched for age and sex, since both factors are well known to correlate with cognitive performance^24,63^. Thus, the sex-specific effects found in the current study regarding cognitive profiles line up with previous studies showing that sex differences exist, and might be of special importance for our society, but are of rather small effect size^10^.

After stratifying the current sample for sex, we again performed an ePCA and obtained different component solutions for each group. While in the male group, three components were preferred (according to the Eigenvalue criterion), females’ cognitive performance was best described by a four-component solution. More importantly, the extracted components differed in their composition, i.e. cognitive tasks involved in the different components. While for the whole group, the first component was composed of heterogeneous but consistently non-verbal functions, verbal as well as non-verbal functions belong to the same component in males, additionally including fluency, memory, attention and executive functions. The second male component contained visual working memory and executive functions and the third component consisted of verbal working memory and executive functions. Relating these results to the observations made regarding task specific differences in processing styles, i.e. global–local hypothesis of sex-differences^11^, one could argue that males’ holistic/global processing style to solve cognitive task, is in line with the current cognitive profile. Males show a quite holistic first component, including attention, executive functions, episodic memory and fluency tasks, hinting at higher interrelations between different cognitive abilities. Furthermore, since executive functions and/or attention depict essential parts in all three components it could be assumed that these functions serve as a higher order executive-attention system that monitors cognitive performance^63–66^. Thus, this would mean that males rely strongly on their attentional and executive functions, e.g. goal-directed planning, monitoring, mental flexibility, to process cognitive tasks belonging to different cognitive domains. In terms of a global way of cognitive processing, males potentially manage cognitive processing using one superordinate system that links different cognitive abilities. Likewise, if these functions decline, a decline of all other cognitive domains follow, as has been stated by theories, such as the frontal executive theory of aging^67^. Investigating females only revealed a different picture compared to both the whole group or males only. Females’ cognitive performance within the functions examined is best decomposed into four cognitive components. In contrast to the males’ first component which was quite heterogeneous including fluency, executive functions and attention, in the females’ cognitive profile visual-verbal fluency and executive functions—attention—built separate components; together with a component composed of visual (working) memory and executive functions and another component dominated by verbal functions including working memory and vocabulary. Thus, females’ cognitive profiles consist of more subsystems as compared to males, with systems including different cognitive functions (i.e. [1] visual (working) memory/[2] fluency/[3] executive functions/[4] verbal (working) memory). Although these functions share covariances, they themselves represent distinct cognitive systems or modules. On the other hand, males might have a superordinate system, i.e. the attentional-executive-fluency-memory component, which includes several cognitive domains, thereby representing a stronger interplay of cognitive functions with a probably superordinate system (i.e. executive functions). Hence, this could be potentially related to a more global processing strategy during cognitive performance, meaning that irrespective of the task (e.g. memory or fluency), males might activate similar cognitive processing strategies. In contrast to that, females would rather choose different processing strategies, depending on the cognitive task, e.g. visual versus verbal working memory. Together, similarly to the global versus local processing at single task level^11,12,15,18,68^, cognitive profiles derived from either males or females seem to be differentially composed along the global vs. local processing dichotomy in the current study.

Furthermore, focussing on the cross-validation model fit values, an additional support for the existence of sex-related cognitive profiles in line with these processing strategies became evident. While applying the female component solution to the female group reveals excellent fit values, the male component solution only reveals acceptable fit values when applied to the male group. These lower fit values might arise from the stronger interconnectedness of different cognitive functions in the male group, which has been shown when comparing correlation strength between males and females (cf Fig. 2). For example, interference is correlated to both, verbal fluency as well as visual spatial short-term memory, which in turn is correlated with figural fluency. As a consequence, a clear division of cognitive functions into different (independent) cognitive domains, might not be possible in the male group. Thus, males’ cognitive abilities seem to be not fully suitable for a modular cognitive structure as compared to females. This again, would be in accordance with global versus local processing styles.

Importantly, the current study investigated an older adult population to examine sex-specific cognitive profiles. This population is of special interest when examining sex differences in cognitive performance and cognitive profiles since previous research has shown that first, sex-differences in cognitive functions remain stable until older ages, and second, pathological conditions with cognitive impairments differ in prevalence between males and females^28,57^. However, research so far, most often includes sex as a covariate of non-interest when assessing cognitive impairment.

Previous studies often showed steeper decline in general cognitive functions in males^1,69^. Similarly, in pathological conditions, such as Parkinson’s disease, males were reported to show a faster decline in cognitive functions^28^. However, when it comes to Alzheimer’s disease, females show a faster decline in memory scores as compared to males^29^. This observation might be related to distinct cognitive profiles in older males and females. If, within the ‘global’ cognitive profile of males, the executive-attentional monitoring system breaks down this would lead to a global decline in cognitive functions. Especially for the aging process, theories such as the prefrontal-executive theory^67^ as well as the processing speed theory^70^ of aging, stating that decreasing executive functions and attention, respectively, predict cognitive decline in a diversity of cognitive functions belonging to different domains^64^. Thus, in males these two theories that try to explain cognitive performance decline during the aging process, would be in line with the current results. On the other hand, if females’ cognitive profiles are rather composed of different cognitive subsystems or modules (thus ‘local’ cognitive profile), impairments within the executive-attentional component would not necessarily lead to an impairment in other cognitive components. Hence, this would rather result in function-specific cognitive decline, e.g. executive impairment. These differences in cognitive profiles might thus serve as a possible explanation for why males show generally steeper decreases in overall cognitive abilities during aging^69^.

### Methodological considerations

The current study has several advantages and disadvantages. While we were able to show that cognitive profiles differ, when investigating males and females independent of each other, it is important to mention that the effects of sex differences are rather of smaller sizes, which becomes obvious when focussing on the differences in terms of intercorrelations between different cognitive tasks. Nevertheless, as stated by Zell et al.^10^, although effect sizes might be small, when investigating sex differences in cognitive performance, these differences might be important to understand cognitive performance differences.

In addition, it has to be mentioned that the current study investigated these cognitive profiles in a sample ranging from 55 to 85 years of age. It might be the case that with increasing age, cognitive profiles change, especially when cognitive impairments arise, e.g. due to pathological conditions. Future studies should investigate this topic, especially using longitudinal data, to show whether cognitive profiles change in the course of the aging process, potentially also with respect to pathological conditions.

Further, it has to be mentioned that the set-up of cognitive profiles is not straightforward. We used a Principal Component Analysis with Varimax rotation method for extracting cognitive components in the two groups and extracted four factors for females and three factors for males, based on the Eigenvalue criterion (cut-off for selection of components being an Eigenvalue > 1). Nevertheless, the fourth Eigenvalue is only slightly above one for females (1.02) and the fourth Eigenvalue is only slightly below 1 (0.97) for males, which both are very close to the cut-off value. Further, the model refinement highly depends on the input data (in this case the cognitive tasks used). Until now, there is no gold standard in this respect. More research is needed to address this important topic.

Finally, the question that arises when observing these differences is which factors might be responsible for the development of sex differences. From previous studies it is known that males and females differ in terms of brain structure and function, which might relate to differences in cognitive processing strategies^71,72^. Furthermore, it has been shown that hormonal differences, but also genetic variations might be related to differences in cognitive and social behavior between the two sexes^73^. Social factors, such as gender role models, significantly influence differences in cognitive performance, which is less pronounced in countries that promote gender equality^74^. Further studies are warranted to examine this question.

## Conclusion

Conclusively, males and females show not only differences in specific cognitive tasks but generally in cognitive profiles across cognitive domains. Males are likely to use a more holistic way of processing, by integrating different cognitive functions to solve specific tasks. This could be, for example, a higher executive control and memory function in a verbal fluency task, which in turn, would result in larger clusters of the same category. Females, on the other hand are likely to process cognitive tasks in smaller, rather domain-specific subsystems. The results showed that older males and females exhibit different cognitive profiles, that are likely to be related to differences in cognitive decline across the aging process. Therefore, the current research stresses the importance to use sex-stratified analyses when assessing cognitive performance. Future research is warranted to extend the current results to pathological conditions, such as Alzheimer’s disease. Furthermore, differences in cognitive profiles might not only be important in basic research but, might also impact clinical prevention programs, i.e. cognitive training.

## Supplementary Information

Supplementary Information
