# Supplementary material for: Cognitive profiles in older males and females
Source: Sci Rep. 2021 Mar 22;11:6524. doi: 10.1038/s41598-021-84134-8 (PMC7985508; doi:10.1038/s41598-021-84134-8)
Supplement: Supplementary file 1 — Supplementary Information [file 41598_2021_84134_MOESM1_ESM.docx]

**Cognitive profiles in older males and females**

**C.Jockwitz^1,2*^, L. Wiersch^3^, J. Stumme^1,2^, S.Caspers^1,2^**

*^1^ Institute of Neuroscience and Medicine (INM-1), Research Centre Jülich, Jülich, Germany*

*^2^ Institute for Anatomy I, Medical Faculty & University Hospital Düsseldorf, Heinrich Heine University Düsseldorf, Düsseldorf, Germany*

*^3^ Institute of Neuroscience and Medicine (INM-7), Research Centre Jülich, Jülich, Germany*

Figure S1: Comparison of the three versus four component solution (left column: three components; right column: four components) for the whole group (a,b), males (c,d) and females (e,f).

SA = Selective Attention, PrcS = Processing Speed, Vc, Vocabulary, PF = Phonemic Fluency, SF = Semantic Fluency, VrSTM = Verbal short-term memory, VrWM = Verbal Working Memory, VsSTM = Visual short-term memory, VsWM = Visual Working Memory, VSS = Visual spatial short-term Memory, FF = Figural Fluency, FM = Figural Memory, EM = Episodic Memory, CS = Concept Shifting, In = Interference, PrbS = Problem Solving

Table S1: Correlation matrix between cognitive performance in males. SA = Selective Attention, PrcS = Processing Speed, Vc, Vocabulary, PF = Phonemic Fluency, SF = Semantic Fluency, VrSTM = Verbal short-term memory, VrWM = Verbal Working Memory, VsSTM = Visual short-term memory, VsWM = Visual Working Memory, VSS = Visual spatial short-term Memory, FF = Figural Fluency, FM = Figural Memory, EM = Episodic Memory, CS = Concept Shifting, In = Interference, PrbS = Problem Solving

| male | SA | PrcS | Vc | PF | SF | VrSTM | VrWM | VsSTM | VsWM | VSS | FF | FM | EM | CS | In | PrbS |
| --- | --- | --- | --- | --- | --- | --- | --- | --- | --- | --- | --- | --- | --- | --- | --- | --- |
| SA | 1 |  |  |  |  |  |  |  |  |  |  |  |  |  |  |  |
| PrcS | .47 | 1 |  |  |  |  |  |  |  |  |  |  |  |  |  |  |
| Vc | .2 | .27 | 1 |  |  |  |  |  |  |  |  |  |  |  |  |  |
| PF | .2 | .26 | .4 | 1 |  |  |  |  |  |  |  |  |  |  |  |  |
| SF | .2 | .22 | .34 | .38 | 1 |  |  |  |  |  |  |  |  |  |  |  |
| VrSTM | .12 | .17 | .24 | .25 | .05 | 1 |  |  |  |  |  |  |  |  |  |  |
| VrWM | .18 | .13 | .2 | .15 | -.03 | .42 | 1 |  |  |  |  |  |  |  |  |  |
| VsSTM | .13 | .2 | -.04 | 0 | .03 | .1 | .22 | 1 |  |  |  |  |  |  |  |  |
| VsWM | .17 | .22 | .08 | .11 | .03 | .16 | .25 | .33 | 1 |  |  |  |  |  |  |  |
| VSS | .26 | .28 | .25 | .16 | .21 | .29 | .22 | .33 | .34 | 1 |  |  |  |  |  |  |
| FF | .39 | .44 | .34 | .26 | .36 | .19 | .13 | .17 | .18 | .41 | 1 |  |  |  |  |  |
| FM | .37 | .38 | .38 | .21 | .27 | .31 | .31 | .22 | .35 | .57 | .44 | 1 |  |  |  |  |
| EM | .22 | .26 | .35 | .24 | .31 | .13 | .27 | .1 | .18 | .29 | .28 | .46 | 1 |  |  |  |
| CS | .3 | .2 | .3 | .19 | .19 | .3 | .31 | .17 | .18 | .3 | .31 | .42 | .3 | 1 |  |  |
| In | .28 | .22 | .24 | .19 | .28 | .18 | .17 | .11 | .17 | .33 | .28 | .39 | .29 | .33 | 1 |  |
| PrbS | .36 | .37 | .4 | .2 | .22 | .35 | .31 | .23 | .33 | .48 | .44 | .54 | .36 | .42 | .37 | 1 |

Table S2: Correlation matrix between cognitive performance in females. SA = Selective Attention, PrcS = Processing Speed, Vc, Vocabulary, PF = Phonemic Fluency, SF = Semantic Fluency, VrSTM = Verbal short-term memory, VrWM = Verbal Working Memory, VsSTM = Visual short-term memory, VsWM = Visual Working Memory, VSS = Visual spatial short-term Memory, FF = Figural Fluency, FM = Figural Memory, EM = Episodic Memory, CS = Concept Shifting, In = Interference, PrbS = Problem Solving

| female | SA | PrcS | Vc | PF | SF | VrSTM | VrWM | VsSTM | VsWM | VSS | FF | FM | EM | CS | In | PrbS |
| --- | --- | --- | --- | --- | --- | --- | --- | --- | --- | --- | --- | --- | --- | --- | --- | --- |
| SA | 1 |  |  |  |  |  |  |  |  |  |  |  |  |  |  |  |
| PrcS | .38 | 1 |  |  |  |  |  |  |  |  |  |  |  |  |  |  |
| Vc | .16 | .13 | 1 |  |  |  |  |  |  |  |  |  |  |  |  |  |
| PF | .21 | .19 | .43 | 1 |  |  |  |  |  |  |  |  |  |  |  |  |
| SF | .22 | .22 | .37 | .5 | 1 |  |  |  |  |  |  |  |  |  |  |  |
| VrSTM | .11 | .14 | .22 | .23 | .17 | 1 |  |  |  |  |  |  |  |  |  |  |
| VrWM | .16 | .18 | .32 | .23 | .17 | .3 | 1 |  |  |  |  |  |  |  |  |  |
| VsSTM | .26 | .23 | .13 | .2 | .17 | .2 | .23 | 1 |  |  |  |  |  |  |  |  |
| VsWM | .25 | .25 | .23 | .18 | .13 | .16 | .19 | .4 | 1 |  |  |  |  |  |  |  |
| VSS | .3 | .32 | .27 | .17 | .24 | .21 | .23 | .35 | .37 | 1 |  |  |  |  |  |  |
| FF | .33 | .29 | .26 | .27 | .39 | .2 | .22 | .2 | .24 | .24 | 1 |  |  |  |  |  |
| FM | .32 | .3 | .39 | .32 | .33 | .23 | .28 | .33 | .4 | .47 | .32 | 1 |  |  |  |  |
| EM | .28 | .21 | .31 | .22 | .32 | .14 | .2 | .14 | .23 | .3 | .2 | .39 | 1 |  |  |  |
| CS | .26 | .2 | .25 | .23 | .16 | .17 | .18 | .21 | .25 | .21 | .23 | .33 | .25 | 1 |  |  |
| In | .23 | .24 | .19 | .1 | .07 | .16 | .2 | .18 | .18 | .2 | .27 | .29 | .24 | .31 | 1 |  |
| PrbS | .31 | .31 | .46 | .39 | .3 | .23 | .37 | .28 | .41 | .43 | .39 | .51 | .31 | .26 | .27 | 1 |

Table S3: Correlation matrix between cognitive performance in the whole group. SA = Selective Attention, PrcS = Processing Speed, Vc, Vocabulary, PF = Phonemic Fluency, SF = Semantic Fluency, VrSTM = Verbal short-term memory, VrWM = Verbal Working Memory, VsSTM = Visual short-term memory, VsWM = Visual Working Memory, VSS = Visual spatial short-term Memory, FF = Figural Fluency, FM = Figural Memory, EM = Episodic Memory, CS = Concept Shifting, In = Interference, PrbS = Problem Solving

| whole | SA | PrcS | Vc | PF | SF | VrSTM | VrWM | VsSTM | VsWM | VSS | FF | FM | EM | CS | In | PrbS |
| --- | --- | --- | --- | --- | --- | --- | --- | --- | --- | --- | --- | --- | --- | --- | --- | --- |
| SA | 1 |  |  |  |  |  |  |  |  |  |  |  |  |  |  |  |
| PrcS | .42 | 1 |  |  |  |  |  |  |  |  |  |  |  |  |  |  |
| Vc | .18 | .21 | 1 |  |  |  |  |  |  |  |  |  |  |  |  |  |
| PF | .2 | .23 | .41 | 1 |  |  |  |  |  |  |  |  |  |  |  |  |
| SF | .21 | .23 | .36 | .44 | 1 |  |  |  |  |  |  |  |  |  |  |  |
| VrSTM | .12 | .15 | .23 | .24 | .11 | 1 |  |  |  |  |  |  |  |  |  |  |
| VrWM | .17 | .15 | .26 | .18 | .06 | .36 | 1 |  |  |  |  |  |  |  |  |  |
| VsSTM | .2 | .21 | .05 | .09 | .09 | .15 | .22 | 1 |  |  |  |  |  |  |  |  |
| VsWM | .21 | .23 | .15 | .13 | .07 | .16 | .22 | .37 | 1 |  |  |  |  |  |  |  |
| VSS | .27 | .28 | .25 | .13 | .2 | .25 | .22 | .35 | .37 | 1 |  |  |  |  |  |  |
| FF | .36 | .37 | .3 | .26 | .37 | .19 | .17 | .18 | .21 | .32 | 1 |  |  |  |  |  |
| FM | .34 | .34 | .38 | .26 | .3 | .27 | .29 | .28 | .37 | .51 | .38 | 1 |  |  |  |  |
| EM | .24 | .25 | .32 | .27 | .32 | .13 | .21 | .09 | .15 | .2 | .22 | .4 | 1 |  |  |  |
| CS | .28 | .21 | .28 | .22 | .18 | .24 | .25 | .18 | .2 | .23 | .27 | .37 | .29 | 1 |  |  |
| In | .25 | .23 | .21 | .16 | .19 | .17 | .18 | .14 | .16 | .24 | .27 | .34 | .29 | .33 | 1 |  |
| PrbS | .33 | .34 | .43 | .28 | .25 | .3 | .34 | .26 | .37 | .45 | .42 | .52 | .3 | .34 | .32 | 1 |

Table S4: Eigenvalues for components in the different groups: whole group, females, males. Eigenvalues > 1 are marked in bold.

| No Comp | Eigenvalues | | |
| --- | --- | --- | --- |
|  | whole | female | male |
| 1 | **4.9371** | **4.9660** | **5.0754** |
| 2 | **1.4797** | **1.3605** | **1.5840** |
| 3 | **1.1873** | **1.0949** | **1.2470** |
| 4 | 0.9904 | **1.0205** | 0.9656 |
| 5 | 0.8922 | 0.9344 | 0.9174 |
| 6 | 0.7953 | 0.8316 | 0.8013 |
| 7 | 0.7560 | 0.7801 | 0.7629 |
| 8 | 0.6892 | 0.7149 | 0.7079 |
| 9 | 0.6567 | 0.6638 | 0.6395 |
| 10 | 0.6377 | 0.6475 | 0.5752 |

Table S5: Component loadings for the component solutions

|  | Whole | | | Males | | | Females | | | |
| --- | --- | --- | --- | --- | --- | --- | --- | --- | --- | --- |
|  | W1 | W2 | W3 | M1 | M2 | M3 | F1 | F2 | F3 | F4 |
| Problem Solving | 0.52 | 0.38 | 0.37 | 0.41 | 0.49 | 0.39 | 0.45 | 0.42 | 0.24 | 0.33 |
| Visual STM | 0.63 | -0.14 | 0.2 | -0.16 | 0.69 | 0.08 | 0.72 | 0 | 0.03 | 0.19 |
| Visual WM | 0.63 | -0.06 | 0.26 | -0.04 | 0.62 | 0.22 | 0.72 | 0.06 | 0.1 | 0.16 |
| Visual Spatial STM | 0.65 | 0.12 | 0.25 | 0.28 | 0.61 | 0.27 | 0.67 | 0.18 | 0.15 | 0.12 |
| Verbal STM | 0.1 | 0.15 | 0.7 | 0.12 | 0.07 | 0.75 | 0.15 | 0.11 | 0.09 | 0.64 |
| Verbal WM | 0.2 | 0.09 | 0.71 | -0.02 | 0.21 | 0.76 | 0.19 | 0.15 | 0.15 | 0.65 |
| Figural Memory | 0.56 | 0.4 | 0.31 | 0.45 | 0.52 | 0.36 | 0.51 | 0.36 | 0.3 | 0.24 |
| Selective Attention | 0.57 | 0.36 | -0.15 | 0.46 | 0.46 | -0.04 | 0.4 | 0.26 | 0.5 | -0.23 |
| Interference | 0.35 | 0.35 | 0.13 | 0.43 | 0.29 | 0.19 | 0.06 | -0.08 | 0.78 | 0.25 |
| Figural Fluency | 0.46 | 0.5 | -0.02 | 0.6 | 0.42 | 0.01 | 0.21 | 0.45 | 0.41 | 0.01 |
| Episodic Memory | 0.18 | 0.56 | 0.15 | 0.49 | 0.19 | 0.29 | 0.17 | 0.39 | 0.41 | 0.07 |
| Phonematic Fluency | -0.03 | 0.68 | 0.23 | 0.61 | -0.14 | 0.26 | 0.09 | 0.73 | 0.02 | 0.24 |
| Semantic Fluency | 0.07 | 0.74 | -0.06 | 0.73 | 0 | -0.1 | 0.1 | 0.83 | 0.05 | -0.01 |
| Processing Speed | 0.56 | 0.38 | -0.14 | 0.49 | 0.48 | -0.06 | 0.44 | 0.21 | 0.42 | -0.19 |
| Concept Shifting | 0.31 | 0.35 | 0.33 | 0.33 | 0.27 | 0.47 | 0.1 | 0.1 | 0.62 | 0.24 |
| Vocabulary | 0.04 | 0.63 | 0.38 | 0.66 | -0.08 | 0.36 | 0.08 | 0.57 | 0.13 | 0.46 |

Table S6: Estimates (using Maximum Likelihood estimation) of Confirmatory factor analysis for the whole group (W1,2,3), males (M1,2,3), and females (F1,2,3)

|  |  | Estimate | SD | z-value | P(>\|z\|) | Std.lv | Std.all |
| --- | --- | --- | --- | --- | --- | --- | --- |
| W1 | Visual Spatial STM | 0.648 | 0.038 | 17.147 | < .001 | 0.648 | 0.648 |
|  | Visual STM | 0.408 | 0.041 | 9.98 | < .001 | 0.408 | 0.408 |
|  | Visual WM | 0.497 | 0.04 | 12.477 | < .001 | 0.497 | 0.497 |
|  | Selective Attention | 0.472 | 0.04 | 11.759 | < .001 | 0.472 | 0.473 |
|  | Figural Memory | 0.63 | 0.05 | 12.726 | < .001 | 0.63 | 0.634 |
|  | Processing Speed | 0.482 | 0.04 | 12.04 | < .001 | 0.482 | 0.482 |
|  | Problem Solving | 0.719 | 0.036 | 19.762 | < .001 | 0.719 | 0.723 |
|  | Figural Fluency | 0.399 | 0.053 | 7.592 | < .001 | 0.399 | 0.4 |
| W2 | Semantic Fluency | 0.63 | 0.041 | 15.396 | < .001 | 0.63 | 0.631 |
|  | Phonemic Fluency | 0.623 | 0.041 | 15.189 | < .001 | 0.623 | 0.623 |
|  | Vocabulary | 0.625 | 0.04 | 15.443 | < .001 | 0.625 | 0.629 |
|  | Episodic Memory | 0.494 | 0.042 | 11.665 | < .001 | 0.494 | 0.494 |
|  | Figural Fluency | 0.244 | 0.055 | 4.418 | < .001 | 0.244 | 0.244 |
|  | Figural Memory | 0.14 | 0.053 | 2.673 | .008 | 0.14 | 0.141 |
| W3 | Verbal WM | 0.619 | 0.051 | 12.159 | < .001 | 0.619 | 0.619 |
|  | Verbal STM | 0.586 | 0.05 | 11.783 | < .001 | 0.586 | 0.587 |
| M1 | Semantic Fluency | 0.457 | 0.056 | 8.127 | < .001 | 0.457 | 0.457 |
|  | Vocabulary | 0.549 | 0.055 | 10.05 | < .001 | 0.549 | 0.55 |
|  | Phonematic Fluency | 0.383 | 0.057 | 6.669 | < .001 | 0.383 | 0.385 |
|  | Figural Fluency | 0.664 | 0.054 | 12.385 | < .001 | 0.664 | 0.665 |
|  | Episodic Memory | 0.588 | 0.056 | 10.593 | < .001 | 0.588 | 0.589 |
|  | Processing Speed | 0.532 | 0.055 | 9.64 | < .001 | 0.532 | 0.532 |
|  | Selective Attention | 0.5 | 0.056 | 8.977 | < .001 | 0.5 | 0.501 |
|  | Figural Memory | 0.386 | 0.086 | 4.467 | < .001 | 0.386 | 0.386 |
|  | Interference | 0.504 | 0.055 | 9.096 | < .001 | 0.504 | 0.505 |
|  | Problem Solving | 0.421 | 0.086 | 4.901 | < .001 | 0.421 | 0.422 |
| M2 | Visual STM | 0.371 | 0.06 | 6.196 | < .001 | 0.371 | 0.372 |
|  | Visual WM | 0.467 | 0.059 | 7.99 | < .001 | 0.467 | 0.468 |
|  | Visual Spatial STM | 0.76 | 0.058 | 13.049 | < .001 | 0.76 | 0.761 |
|  | Figural Memory | 0.472 | 0.087 | 5.424 | < .001 | 0.472 | 0.473 |
|  | Problem Solving | 0.359 | 0.087 | 4.142 | < .001 | 0.359 | 0.36 |
| M3 | Verbal WM | 0.463 | 0.063 | 7.338 | < .001 | 0.463 | 0.464 |
|  | Verbal STM | 0.468 | 0.063 | 7.424 | < .001 | 0.468 | 0.469 |
|  | Concept Shifting | 0.653 | 0.064 | 10.138 | < .001 | 0.653 | 0.654 |
| F1 | Visual STM | 0.466 | 0.057 | 8.187 | < .001 | 0.466 | 0.467 |
|  | Visual WM | 0.554 | 0.055 | 10.007 | < .001 | 0.554 | 0.554 |
|  | Visual Spatial STM | 0.629 | 0.054 | 11.722 | < .001 | 0.629 | 0.63 |
|  | Figural Memory | 0.735 | 0.052 | 14.216 | < .001 | 0.735 | 0.736 |
|  | Problem Solving | 0.574 | 0.066 | 8.71 | < .001 | 0.574 | 0.575 |
| F2 | Semantic Fluency | 0.673 | 0.058 | 11.699 | < .001 | 0.673 | 0.676 |
|  | Phonemic Fluency | 0.717 | 0.058 | 12.341 | < .001 | 0.717 | 0.718 |
|  | Vocabulary | 0.39 | 0.087 | 4.501 | < .001 | 0.39 | 0.391 |
|  | Figural Fluency | 0.267 | 0.076 | 3.487 | < .001 | 0.267 | 0.267 |
|  | Problem Solving | 0.224 | 0.069 | 3.254 | 0.001 | 0.224 | 0.224 |
| F3 | Interference | 0.449 | 0.06 | 7.522 | < .001 | 0.449 | 0.45 |
|  | Concept Shifting | 0.466 | 0.059 | 7.838 | < .001 | 0.466 | 0.466 |
|  | Selective Attention | 0.571 | 0.057 | 9.936 | < .001 | 0.571 | 0.571 |
|  | Processing Speed | 0.529 | 0.058 | 9.112 | < .001 | 0.529 | 0.529 |
|  | Figural Fluency | 0.365 | 0.075 | 4.887 | < .001 | 0.365 | 0.366 |
|  | Episodic Memory | 0.504 | 0.058 | 8.671 | < .001 | 0.504 | 0.505 |
| F4 | Verbal WM | 0.588 | 0.07 | 8.425 | < .001 | 0.588 | 0.589 |
|  | Verbal STM | 0.466 | 0.066 | 7.043 | < .001 | 0.466 | 0.467 |
|  | Vocabulary | 0.335 | 0.089 | 3.771 | < .001 | 0.335 | 0.336 |

Table S7: Covariances between Variables for the different models

|  |  | Estimate | Std.Err | z-value | P(>\|z\|) |
| --- | --- | --- | --- | --- | --- |
| Whole | Visual STM ~ Visual WM | 0.164 | 0.033 | 4.898 | < .001 |
|  | Selective Attention ~ Processing Speed | 0.195 | 0.033 | 5.85 | < .001 |
|  | Figural Memory ~ Episodic Memory | 0.11 | 0.028 | 3.918 | < .001 |
|  | Problem Solving ~ Vocabulary | 0.119 | 0.026 | 4.533 | < .001 |
|  | W1 ~ W2 | 0.59 | 0.041 | 14.224 | < .001 |
|  | W1 ~ W3 | 0.628 | 0.049 | 12.901 | < .001 |
|  | W2 ~ W3 | 0.47 | 0.057 | 8.269 | < .001 |
| Males | Semantic Fluency ~ Phonematc Fluency | 0.183 | 0.046 | 3.956 | < .001 |
|  | Vocabulary ~ Phonemic Fluency | 0.171 | 0.044 | 3.842 | < .001 |
|  | Figural Fluency ~ Episodic Memory | -0.113 | 0.04 | -2.855 | 0.004 |
|  | Processing Speed ~ Selective Attention | 0.202 | 0.045 | 4.453 | < .001 |
|  | Visual STM ~ Visual WM | 0.151 | 0.049 | 3.084 | 0.002 |
|  | Verbal WM ~ Verbal STM | 0.198 | 0.052 | 3.791 | < .001 |
|  | M1 ~ M2 | 0.667 | 0.061 | 10.935 | < .001 |
|  | M1 ~ M3 | 0.746 | 0.064 | 11.607 | < .001 |
|  | M2 ~ M3 | 0.722 | 0.073 | 9.931 | < .001 |
| Females | Visual STM ~ Visual WM | 0.139 | 0.045 | 3.076 | 0.002 |
|  | Semantic Fluency ~ Episodic Memory | 0.111 | 0.041 | 2.685 | 0.007 |
|  | Interference ~ Concept Shiftng | 0.102 | 0.048 | 2.123 | 0.034 |
|  | F1 ~ F2 | 0.535 | 0.066 | 8.144 | < .001 |
|  | F1 ~ F3 | 0.84 | 0.045 | 18.62 | < .001 |
|  | F1 ~ F4 | 0.718 | 0.072 | 9.902 | < .001 |
|  | F2 ~ F3 | 0.528 | 0.07 | 7.499 | < .001 |
|  | F2 ~ F4 | 0.545 | 0.092 | 5.932 | < .001 |
|  | F3 ~ F4 | 0.593 | 0.085 | 6.982 | < .001 |

Table 8: Measurement invariance of the whole group males with parameter estimates for males and females separately.

|  |  | MAles | | | | Females | | | |
| --- | --- | --- | --- | --- | --- | --- | --- | --- | --- |
|  |  | Estimate | SD | z | P | Estimate | sd | z | P |
| W1 | Visual Spatial STM | 0.612 | 0.053 | 11.487 | < .001 | 0.667 | 0.05 | 13.246 | < .001 |
|  | Visual STM | 0.483 | 0.059 | 8.216 | < .001 | 0.323 | 0.056 | 5.767 | < .001 |
|  | Visual WM | 0.534 | 0.055 | 9.779 | < .001 | 0.442 | 0.057 | 7.738 | < .001 |
|  | Selective Attention | 0.484 | 0.058 | 8.337 | < .001 | 0.465 | 0.055 | 8.455 | < .001 |
|  | Figural Memory | 0.579 | 0.083 | 6.995 | < .001 | 0.676 | 0.071 | 9.58 | < .001 |
|  | Processing Speed | 0.44 | 0.054 | 8.168 | < .001 | 0.537 | 0.058 | 9.206 | < .001 |
|  | Problem Solving | 0.687 | 0.05 | 13.854 | < .001 | 0.75 | 0.053 | 14.124 | < .001 |
|  | Figural Fluency | 0.326 | 0.087 | 3.767 | < .001 | 0.445 | 0.075 | 5.942 | < .001 |
| W2 | Semantic Fluncy | 0.638 | 0.053 | 12.012 | < .001 | 0.596 | 0.062 | 9.693 | < .001 |
|  | Phonemic Fluency | 0.649 | 0.056 | 11.601 | < .001 | 0.553 | 0.058 | 9.526 | < .001 |
|  | Vocabulary | 0.624 | 0.057 | 10.952 | < .001 | 0.638 | 0.057 | 11.225 | < .001 |
|  | Episodic Memory | 0.442 | 0.056 | 7.93 | < .001 | 0.503 | 0.056 | 8.945 | < .001 |
|  | Figural Fluency | 0.232 | 0.089 | 2.603 | 0.009 | 0.273 | 0.08 | 3.42 | 0.001 |
|  | Figural Memory | 0.152 | 0.086 | 1.775 | 0.076 | 0.108 | 0.076 | 1.412 | 0.158 |
| W3 | Verbal WM | 0.585 | 0.073 | 8.057 | < .001 | 0.633 | 0.072 | 8.842 | < .001 |
|  | Verbal STM | 0.472 | 0.066 | 7.174 | < .001 | 0.709 | 0.075 | 9.399 | < .001 |
